# Australian Football Skill-Based Assessments: A Proposed Model for Future Research

**DOI:** 10.3389/fpsyg.2019.00429

**Published:** 2019-02-26

**Authors:** Nathan Bonney, Jason Berry, Kevin Ball, Paul Larkin

**Affiliations:** ^1^Institute for Health and Sport, Victoria University, Melbourne, VIC, Australia; ^2^IMG Academy, Bradenton, FL, United States; ^3^Maribyrnong Sports Academy, Melbourne, VIC, Australia

**Keywords:** talent identification, Australian Football, representative learning design, skill assessment, kicking, motor competence, development, performance

## Abstract

Identifying sporting talent remains a difficult task due to the complex nature of sport. Technical skill assessments are used throughout the talent pathway to monitor athletes in an attempt to more effectively predict future performance. These assessments, however, largely focus on the isolated execution of key skills devoid of any game context. When assessments are representative of match-play and applied in a setting where all four components of competition (i.e., technical, tactical, physiological, and psychological) are assessed within an integrated approach, prediction of talent is more likely to be successful. This article explores the current talent identification technical skill assessments, with a particular focus on Australian Football, and proposes a 5-level performance assessment model for athlete assessment. The model separates technical game skill on a continuum from Level-1 (i.e., laboratory analysis) to Level-5 (i.e., match-play). These levels, using the assumptions of the expert performance model and representative learning design theory, incorporate a step-wise progression of performance demands to more closely represent match-play conditions. The proposed model will provide researchers and practitioners with a structured framework to consider when assessing, or developing, new assessments of technical game-based skill.

## Introduction

Talent is a multi-dimensional concept and requires the effective and efficient organization of an individual’s technical, tactical, physiological, and psychological competencies to be applied in a method that meets the requirements of both the environment and the sporting situation (Abbott et al., 2005; Vaeyens et al., 2008). Talent identification programs endeavor to discover this “talent” in individuals with the greatest potential to respond to a training intervention and reach the highest level in their chosen sport (Hoare and Warr, 2000; Abbott and Collins, 2004). The ability to identify talented players in team sports is not only a financially rewarding business but a key component of future winning teams and long term success (Gee et al., 2010; Larkin and Reeves, 2018).

Identifying sporting talent remains a complex task due to the evolving nature of sport and the limited number of athletes selected to play at the elite level (Simonton, 1999; Honer and Votteler, 2016). Australian Football (AF) talent scouts consider a variety of subjective measures (such as the technical, tactical, physiological, and psychological components) to inform their decisions. These opinions are then combined with objective isolated skill data (e.g., the AF kicking efficiency test) in an attempt to more effectively predict future performance. Ericsson and Smith (1991) created the expert performance approach (EPA) which entailed three stages. The first involves observation of performance *in situ* to identify key components that can be reproduced and assessed in the laboratory. The second stage examines these performances within field-based assessments (i.e., AF kicking efficiency test) in an attempt to understand the factors that contribute to expert performance. The final stage involves efforts to detail the adaptive learning and explicit acquisition processes relevant to the development of expertise, with potential implications for practice and instruction.

The term representative learning design has more recently been discussed in the literature (Krause et al., 2019) and should be considered within the second and third stage of the EPA. Representative learning design (RLD) is a framework that assesses how closely information provided in a task is representative of the specific performance context (Krause et al., 2019). To increase how representative a task is there needs to be functional coupling between perception and action processes, an adequate amount of informational characteristics from within the competitive environment, and consider the interrelating constraints on movement characteristics (Pinder et al., 2011, 2015). For an assessment to replicate competition it must have functionality (i.e., the degree to which a player can use the same informational sources present during competition) and action fidelity (i.e., the degree to which a player’s movements replicate competition) (Stoffregen et al., 2003; Pinder et al., 2011). Krause et al. (2018) used the RLD framework to design an assessment tool to assess and enhance tennis practice session designs to maximize the potential for skill development to transfer into match-play (Krause et al., 2019). The authors found when comparing practice sessions to match play of junior tennis players, practice tasks are not representative of the shot and movement characteristics typical of match play. Overall, this study highlights the importance of the first stage in the EPA model, the identification of key components, in combination with the RLD framework when designing and assessing tasks.

Talent identification programs have generated a series of discussions regarding their value, with some authors questioning their use and predictability during athlete development (Pankhurst et al., 2013; Honer and Votteler, 2016); where others have used these programs with success (Hoare and Warr, 2000). Whilst these debates are warranted, talent identification programs are well ingrained in elite sport and should aim to identify promising athletes from a multidisciplinary approach rather than a reductionist approach. Where tasks are reductionist in approach, they are performed in a controlled environment where unidimensional components (i.e., speed) are assessed in isolation from the performance context, and may not have enough representation to enhance learning in specific sports (Davids et al., 2013b).

The challenge for talent identification and development (TID) programs is not in identifying current talent, but rather in classifying what factors will restrict the development of talent over time. Whilst there are numerous papers identifying current differences in higher and lesser skilled AF players (Veale et al., 2010; Woods et al., 2017), there are only limited attempts at identifying factors underpinning development across the AF pathway (Farrow et al., 2017; Gastin et al., 2017). Further, team sport talent identification programs, focussed on isolated skill development independent of the game environment, may lack the identification of key components such as decision making, game tempo adjustment, and tactical awareness (Burgess and Naughton, 2010). This is supported by Hoare and Warr (2000) who suggest objective assessments that measure tactical and technical awareness are needed as many players are strong athletically but lack these crucial components.

The aim of this paper is to review current Australian Football technical skill assessments, whilst considering the expert performance model and representative learning design theory, to develop a structured framework for practitioners to consider when assessing, or developing, new assessments of technical game based skill. This article applies these foundation concepts to exploring AF skill assessment. A 5-level performance assessment model (PAM) is proposed, attempting to order skill assessments along the performance continuum.

## Methods

The PAM model was developed from an extensive search of the AF literature using the Preferred Reporting Reviews and Meta-Analyses (PRISMA) statement as a guideline (Moher et al., 2009). Studies were included in the final review if they contained the following: (1) AF kicking proficiency test; (2) AF kicking test assessment; (3) AF kicking proficiency. The search strategy commenced with electronic database searches in SPORTDiscus, PubMed, and Google Scholar. Further studies were then examined from secondary sources such as the reference list of articles found from the initial search (Robertson et al., 2014). Search terms were limited to *Australian Football*, *Australian Football League*, *kicking*, *small-sided game*, *skill assessment*, and *skill test*. In total, 282 relevant studies were returned with 19 studies examining the technical skill of the AF drop-punt kick. Ten studies examined the drop-punt from a biomechanical perspective, seven investigated the technical skill from either a performance perspective, for talent identification purposes, or for the classification of playing position. Only two papers investigated the reliability and validity of the current AF kicking test (see Table 1).

**Table 1:** Australian Football kicking test and proficiency investigation papers.

Author(s)	Year	Participants	Investigation
AF kicking test investigations			
^∗^Cripps et al.	2015	121 Sub-elite AFL Players	Inter-rater reliability and validity of AFL kicking and handball tests
^∗^Woods et al.	2015	25 elite U18 AF players and 25 non-state AF players	The use of skill tests to predict status in junior AF
AF kicking proficiency investigations			
^∗^Cripps et al.	2017	282 U16 AF State Academy	The biological maturity, anthropometric, physical, and technical assessment of talent identified AF players
^∗^Gastin et al.	2017	156 amateur 10–15 year old’s	Age related differences in maturity, physical fitness, match running performance, and skill execution proficiency
Heasman et al.	2008	22 AFL games	Development and validation of a player impact ranking system in AF
Joseph et al.	2017	24 elite U18 AF players	The relationship between repeated kicking performance and maximal aerobic capacity
Tangalos et al.	2015	156 amateur 10–15 year old’s	The relationship between fitness, skill and player performance
^∗^Woods et al.	2016	42 talented and 42 non-talent identified U18 AF players	The application of a multi-dimensional assessment approach to talent identification in AF
^∗^Woods et al.	2018	211 U18 state representatives	Classification of playing position in elite junior AF using technical skill indicators


*^∗^Studies that included both kicking and handballing.*

## 5-Level Performance Assessment Model

**FIGURE 1:**
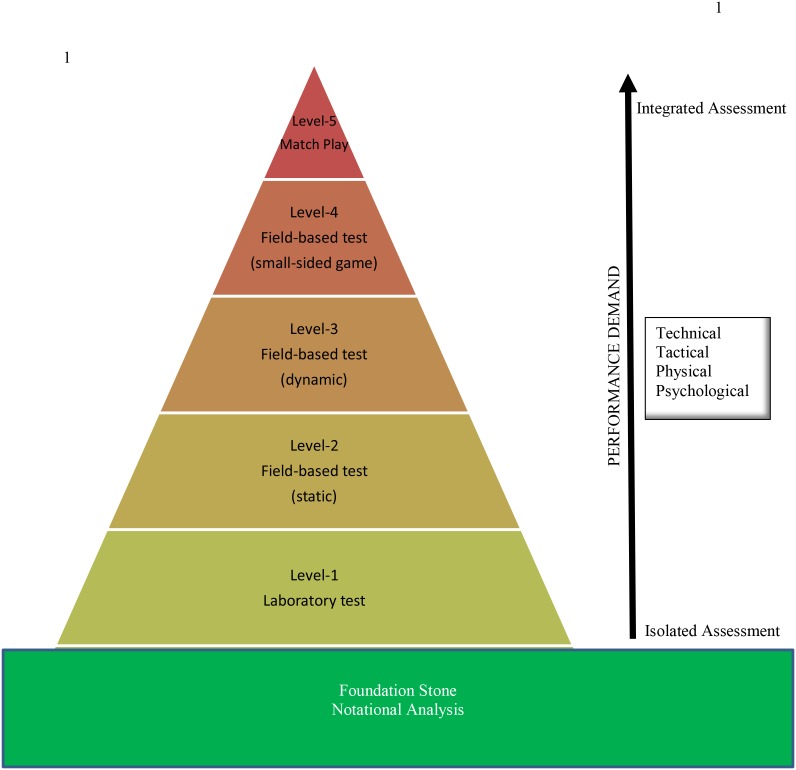
The 5-Level Performance Assessment Model for team-sports.

During match-play all four components of performance (i.e., technical, tactical, physical, and psychological) are required to work in unison whilst the highest demand of intensity/pressure is being placed upon them. The 5-level PAM provides a progression of skill assessment from a performance demand perspective and how representative the assessment is to measure technical skills. At the base of the model is the notational analysis, which is the foundation stone for the PAM. Notational analysis identifies key skills and actions performed within the competitive environment. It is a technique for observing performance and recording the frequencies of these events. As such, in the PAM model, key in-game skills would be notionally analyzed and assessed using the appropriate level on the PAM. Accordingly, the 5-Level PAM proposes match-play is the ultimate level of assessment and resides at the highest point of the performance continuum at Level-5 (see Figure 1).

### Foundation Stone: Notational Analysis

This stone is an application of the first stage of the EPA by identifying key components from match-play to be assessed. Notational analysis from actual game performance is needed to determine the key skill-based outcomes such as technical performance skills. For example, this level may identify ball possession duration, kick distance or locomotion whilst kicking the ball. Without this level of analysis, assessment selection would remain largely subjective and potentially inaccurate.

In AF, notational analysis identified two critical skills to pass and score – handballing and kicking (Sullivan et al., 2014a; Kempton et al., 2015). Although handballing is an effective method to pass the ball (Parrington et al., 2013), in elite junior AF kicking is used more frequently (Woods et al., 2018) and is the only method by which a goal can be scored. The ability to proficiently kick the ball in a game situation (i.e., pass to the intended target or score) is a critical factor, with the drop punt the most commonly used kick in AF (Ball, 2008). Robertson et al. (2015) highlighted the importance of kicking in one AFL team over a 2-year period. Winning teams had more kicks and were able to use the ball more efficiently to both pass the ball and to score. This study revealed teams who had more kicks and greater goal conversion (>4.2%) than their opposition won on 49 of 54 occasions (Robertson et al., 2015). Although kicking is a very specific skill in AF and has been associated with team success, from a talent identification perspective it has been poorly tested and measured in comparison to other AF performance variables (e.g., speed without the ball) (Woods et al., 2015b).

The distance a kick travels is another important consideration. Long kicks (i.e., >40 m) have been associated with a team’s impact score (Heasman et al., 2008) as they create more opportunities for the kicker to score, pass and are harder to defend (Ball, 2008). However, they are generally delivered to a contest (i.e., where several players for each team compete for the ball) where retaining possession becomes more difficult (Appleby and Dawson, 2002). In comparison, Appleby and Dawson (2002) noted short kicks (i.e., <40 m) are commonly delivered to teammates who have moved away from their opponent and are by themselves. This reduces the amount of time the ball spends in the air, reducing the opportunity for opponents to intercept the ball and increasing a team’s effectiveness at retaining possession of the ball (Appleby and Dawson, 2002).

The analysis of skill effectiveness has been used to differentiate between first round and later round draft picks. Findings have shown players drafted from U18 level to elite AF in the first and second rounds had more kicks, effective disposals, contested possessions, and contested marks in the U18 National Championships than athletes selected later in the draft (Woods et al., 2016). Furthermore, drafted players from the Championships, were able to deliver the ball more times within the attacking 50 m zone than non-drafted players (Woods et al., 2015a). Unfortunately, this study did not specify whether these kicks were effective or not, but as this criterion lead to being drafted it is reasonable to assume they were effective. Finally, delivering the ball inside the 50 m zone was deemed more influential to talent scouts than rebound 50’s (a player removing the ball from their defensive 50 m zone) as these kicks lead to scoring situations (Woods et al., 2015a). Notational analysis therefore, is the foundation for any research investigating key aspects of a performance environment. By using this process key performance skills which warrant further investigation can be identified.

### Level-1: Laboratory Test

Laboratory based assessments are performed under well controlled environments and provide highly accurate outcomes (Henriksson et al., 2016). Using the RLD framework, this level would have low representation and therefore is positioned the furthest away from in-game performance. Traditionally, researchers have used laboratory-based tasks in an attempt to reliably capture an aspect of performance which inadvertently separates perception from action (Farrow et al., 2017). In AF, these tests have been largely used to assess, speed and anthropometrics (Pyne et al., 2005; Veale et al., 2008) and to a lesser extent, tactical capabilities (decision making abilities) (Breed et al., 2018) and technical characteristics (biomechanical assessments to analyze key components of the moving body segments to produce a skill) (Dichiera et al., 2006).

Level-1 assessments, such as maximal oxygen uptake (VO_2_ max) (Lorenzen et al., 2009), are an important consideration in AF. On average, AFL players are covering just under 13,000 m per game with over 3000 m being performed at high intensity (between 4.17 and 10.00 ms^-1^) (Aughey, 2010; Coutts et al., 2010). Additionally, this level would be appropriate for identifying particular biomechanical movement patterns, such as the effect lower limb joint angles have on kicking accuracy (Dichiera et al., 2006). Whilst these assessments do provide valuable feedback for coaches and participants (e.g., they can provide appropriate intervention strategies) they do contain certain limitations. Laboratory assessments do not ascertain how proficient a player is with their skill execution under match conditions and they may not consider a player changing their kinematic movements to compensate for any deficiencies they may have when executing a skill (Royal et al., 2006; Coventry et al., 2015).

### Level-2: Field-Based Test (Static)

Level-2 on the performance continuum explores skill execution through field-based testing within a static environment (i.e., AF kicking efficiency test). Although this level is more representative than laboratory tests, it largely focuses on the isolated technical element of key skills required in AF (i.e., the technical ability to kick the ball with limited presence of the physiological, tactical and psychological demands of AF). As such, this test would remain a low rating for representativeness, positioned one level higher on the continuum. Biomechanical researchers have used this level to obtain a more natural assessment of kicking actions. For example, Ball (2008) assessed the isolated technical elements of distance kicking of 28 AFL players on the ground where players trained and played. It was noted greater foot speeds and shank angular velocities, with an increase in last step length, were required to deliver the ball further (Ball, 2008). In a small study investigating the goal-kicking accuracy in two junior AF players, using an inertial measurement system, it was noted the participants had individual differences with goal kicking accuracy suggesting goal kicking assessment in junior athletes require an individual-based analysis (Blair et al., 2017).

In an attempt to more accurately identify and assess talent the Australian Football League (AFL) introduced a draft combine. This combine occurs annually with potential draftees invited to complete a testing battery of standardized technical (e.g., kicking efficiency test), physical (e.g., 2 km time trial), psychological (e.g., personality test), and medical tests (e.g., eye test) conducted over a 4-day period. These measures are then combined with talent scouts subjective opinions of in-game performance to help classify, identify and select players (Burgess et al., 2012). While there are six physical tests, measuring speed (20 m sprint), change of direction (agility test), running endurance (Yo-Yo test and 2 km time trial), and power (standing vertical jump, running vertical jump), currently, there are only two technical skill tests used in the AFL Draft Combine: a goal kicking test and a kicking efficiency test.

The goal kicking test involves players having five shots at goal (i.e., two set shots, two snap shots, and one on the run) within a 70 s period. The kicking efficiency test involves players taking six kicks from a designated distance (i.e., two at 20 m, two at 30 m, and two at 40 m) to a pre-determined location in a random order. Whilst there has been extensive use of the tests at the AFL Draft Combine, there has been minimal research conducted on the use of these AF technical skill assessments. Cripps et al. (2015) indicated the kicking efficiency test is appropriate for assessing kicking in a static environment (i.e., a setting where the skill is performed in an isolated manner, absent of opposition in a relatively predictable environment), whilst also being able to provide feedback to developing athletes regarding dominant and non-dominant disposals over a range of distances. Despite these findings, Cripps et al. (2015) concluded more research is required to determine if the test can also distinguish between higher and lower skilled players and whether kicking ability changes with age. In contrast Woods et al. (2015b) assessed 50 U18 male players (25 state representatives and 25 non-state representatives) and found when kicking accuracy and ball speed were combined, playing status was able to be predicted.

Skill assessment is an important consideration and when combined with physiological competencies, the ability to predict future individual success was increased (Tangalos et al., 2015). The use of technical skill testing to predict performance has been effective in team and individual sports such as handball (Lidor et al., 2005), soccer (Ali et al., 2008), and rugby (Gabbett et al., 2011a). It has been suggested a player’s proficiency in skill execution can potentially influence playing performance and therefore team performance to a larger extent (Gabbett and Ryan, 2009; Gabbett et al., 2011b). This not only highlights the importance of skill in regard to game influence and draft selection, but also emphasizes the importance of further skill development and how it may contribute to match performance (through maintaining possession and effectively delivering the ball) and player development.

Whilst the technical and physiological tests do provide some valuable information (such as the potential to identify skill strengths and weaknesses), they are predominantly performed in a relatively closed setting, separating the skill from the demands of match performance. Research on the kicking efficiency test indicates no significant correlation between coaches’ perceptions of skill and kicking test scores (*r* = -0.13, *P* = 0.75). Construct validity for the test was shown to be poor, however; the kicking test did demonstrate partial content validity and a strong inter-rater reliability score (ICC = 0.96, *P* < 0.01) (Cripps et al., 2015).

Overall, Level-2 assessments are more practical to implement (as they are easier to set up and conduct than laboratory tests), making them not only appropriate at the elite level but also at the sub-elite level. Although Level-2 is accurate at identifying specific body segment skill movements, these movement patterns are done in isolation of game performance demands. This approach has been suggested as one reason as to why talent identification (TID) programs are not effective (Unnithan et al., 2012; Pinder et al., 2013) and why future research should move forward from this approach and include more multidimensional aspects of performance (Pearson et al., 2006). Although this approach would be more complex to develop than univariate assessment, they are more dynamic and may better capture the nuances of talent and how it evolves across development (Johnston et al., 2018). An additional level is therefore required where the skill is executed in a more representative assessment.

### Level-3: Field-Based Test (Dynamic)

Field-based dynamic tests are a further progression along the representation and stability of the environment whilst remaining structured. Where Level-2 tests are pre-planned, Level-3 tests are more open and match-like. They require a minimum of three match-specific components to be integrated at the one time (e.g., technical, tactical, and physiological) and demand a higher level of performance intensity than field-based static tests thereby making them a medium representation of match-play and positioned one level higher than the previous two levels. In ecological dynamics, it has been discussed how the continuous performer-environment interaction is critical to making effective decisions and organizing their actions during performance (Brunswick, 1956; Gisbon, 1979; Travassos et al., 2012).

The process of perception-action coupling occurs throughout Level-3 to facilitate behavior based on the visual information available in the performance environment. Within team sport match-play, successful actions of athletes will vary due to the unpredictable environmental elements and constraints (e.g., opposition movement). Therefore, movements are largely anticipatory in nature based upon key information from their actions and the external environment (Araujo et al., 2006). When assessing the anticipatory visual cues of 25 tennis players (13 skilled and 12 novice) it was found skilled players were more accurate with live and video displays (but not with point-light displays) than novices (Shim et al., 2005). This highlights the importance of information presented to be representative of the performance environment as stimuli presence or absence may shape player behavior (Greenwood et al., 2016).

For example, the use of bowling machines in cricket practice have enabled players to rehearse their physical actions to be trained, however, their use is limited due to the lack of perceptual stimulus displayed. When there is a lack of perceptual stimulus (i.e., bowlers arm, hand, hip, trunk movement) players re-organize their coordination and timing, leading to movement patterns which are not representative of match-play (i.e., peak height of back swing was higher and drive initiation of the downswing occurred earlier and lasted longer when batting against a machine) (Renshaw et al., 2007). Although these studies would suggest human opposition is the preferred option, it is important to be mindful that humans may be more inconsistent, not as fast and may not be able to produce as much volume thereby limiting the amount of quality practice a player has. Careful consideration therefore needs to be applied to designing tests for athletes to complete as there appears to be a continuum as to how representative a test can be.

To the author’s knowledge, there have been no published articles exploring the use of field-based dynamic tests (i.e., Level-3) for the purpose of talent identification in AF. This is an important consideration as in a review of TID in male soccer is was discussed how the combination of a player’s technical and tactical skills in combination with their anthropometric and physiological characteristics is a complex relationship requiring careful attention (Sarmento et al., 2018). This relationship should be considered according to the age, maturational status and specific playing position of each player to avoid discriminating against younger or late-maturing players and the effect these may have on performance capabilities (Sarmento et al., 2018). Tangalos et al. (2015) investigated the relationships between indices of fitness and skill on player performance in 10–15 year old amateur AF players. The author’s found when skill (coach rating) and fitness (20 m shuttle test) were combined, there is a good correlation with the number of disposals an athlete will achieve as well as the number of effective disposals (Tangalos et al., 2015). As the main purpose of a performance test is to demonstrate how that test relates to the competitive environment (Tangalos et al., 2015), researchers need to understand the dynamics of AF and the technical elements. To achieve this, the notational analysis gathered at the Foundation Stone could be used to identify performance specific movement patterns, skill executions and physical demands allowing critical skills to be identified within the context they are performed. For example, identifying how a player obtains the ball, what movement patterns they perform with the ball and how they deliver the ball could then be applied to a dynamic skill test, enabling key factors such as skill executions and movement patterns to be combined together.

The ability to identify the physical and informational constraints from the environment and use opportunities for actions to achieve performance goals (Davids et al., 2013b) is a critical skill that talent scouts are looking for in recruits (Woods et al., 2015a). As such, Level-3 may assist talent scouts in identifying players who are able to do this. In sports like track and field, cricket, and gymnastics, athletes use perceptual variables to regulate their approach to performing the task (i.e., in cricket athletes use the umpire as a way of ascertaining depth perception and to regulate their gait during run up) (Greenwood et al., 2012). Elite coaches’ are also aware of the importance of task constraints in learning design and use non-linear pedagogy to design training around the individual athletes constraints (i.e., their physical, physiological, cognitive, and emotional characteristics) to allow the athlete to solve the performance problem (Greenwood et al., 2012).

Overall, Level-3 contains a more integrated approach of match-play components and a higher requirement from the performance demands (i.e., pressure) than Level-2. The skill execution is assessed with an outcome focus (i.e., kick effectiveness) rather than a performance focus (i.e., the mechanics of the kicking action). When investigating the kinematic effects of a short term fatigue protocol on drop-punt kicking, it was concluded players are able to make kinematic adaptations in order to maintain foot speed while punting for maximal distance (Coventry et al., 2015). Therefore, a player at this level may be effective in their delivery of the ball, however, their mechanics may alter from the preferred technique. This assessment result could potentially be used as a way of determining a player’s ability to execute a skill under particular constraints. A limitation of Level-3 is the absence of opposition and the ability to assess how proficient a player is with their skill execution in a more open and dynamic playing environment. Therefore, a fourth level is required where this can be addressed.

### Level-4: Field-Based Test (Small-Sided Game)

Level-4 is the implementation of field-based small sided games, where all four components of performance are assessed at the one time. This integrated approach of the components enables this test to be high in representation and therefore positioned one level below match-play assessment. Field-hockey coaches have noted that whilst technique is important, so too was practicing in a tactical context where match-play is simulated, as the latter improved players tactical understanding, decision making ability and their understanding of player patterns (Slade, 2015). The absence of live opponents in the current AF skill tests may alter the perceptual cues available to the performer and consequently the performer may use alternative, non-match like movement patterns, leading to an unreliable evaluation of that particular skill performance (Roca and Williams, 2016). In AF there is not one typical stimulus that a player is going to react to (i.e., the umpire blowing their whistle) but a continuous flow of stimuli from the environment that needs to be perceived and responded to (Davids et al., 2005). It has been discussed that a flaw in sports science research is the inability to accurately sample the perceptual variables of performance environments in which skilled athletes operate (Dicks et al., 2009).

When contemplating talent identification in AF, it is apparent more sport specific research is required to obtain clarity on the interconnecting components. Reviews such as the one conducted by Robinson et al. (2018) have highlighted the high level of variability in the elements separating higher and lesser skilled players. A possible suggestion to achieving greater continuity is to have studies based on sound theoretical principles and valid research designs (Bergkamp et al., 2018). New assessments should consider the interacting constraints, movement behaviors, contain adequate environmental variables and ensure the functional coupling between perception and action processes (Pinder et al., 2011). Additionally, they should challenge athletes to make accurate and timely decisions whilst executing the skill under some level of fatigue (Dawson et al., 2004). It is therefore evident a significant gap exists within the AFL talent assessment procedures.

Small-sided games are modified games played on reduced ground areas, often using adapted rules and involve smaller number of players than traditional games (Hill-Haas et al., 2011). The use of small-sided games (SSGs) at Level-4 could be appropriate as they replicate movement constraints (i.e., pressure when kicking the ball, locomotor patterns), information variables from the specific environment and the functional coupling between perception and action processes from competition. Furthermore, the goals in the assessment context (i.e., kicking efficiency) are based on comparable information (i.e., decision making) to the performance environment (Pinder et al., 2011; Loader et al., 2012). When a representative environment allows athletes to display their tactical understanding and their ability to make timely and accurate decisions, combined with their ability to proficiently execute game related skills, players can be accurately identified as either higher skilled or lesser skilled (Oslin et al., 1998; Davids et al., 2013a).

Match-play occurs within an unstable, dynamic and unpredictable environment. Traditional assessment methods (such as those discussed at Level-1 and Level-2), have a tendency to isolate the key components of technical, tactical, physiological and psychological competencies, thereby making the movement patterns more predictable and consequently limiting their application to match-play. There is a strong need for coaches to develop activities where these components are more interconnected whilst replicating the most intense contact demands of competition without a decrease in running performance (Johnston et al., 2015). The use of SSGs and practice matches as a way of developing skill and selecting team members is a well-established concept that most, if not all, sports utilize (Hoare and Warr, 2000; Gabbett, 2009). Team sport coaches of rugby (Gabbett et al., 2012b), soccer (Hill-Haas et al., 2011), and AF (Davies et al., 2013) have implemented small sided games as part of their training regimen in an attempt to develop decision making (O’Connor et al., 2017), skill execution (Klusemann et al., 2012), and tactical awareness skills (Chatzopoulos et al., 2006). This style of training creates an environment where the interaction between players are constantly changing in a dynamic manner thereby creating opportunities to challenge the athlete to make timely decisions whilst efficiently disposing of the ball in a simulated match environment (Davids et al., 2013a).

The size of the small-sided game perimeter has varied within the literature due to the focus being on specific fitness qualities rather than talent assessment. In AF, playing areas such as 30 m × 20 m, 45 m × 30 m, 23.2 m × 20 m, 30 m × 20 m have all been used to compare the agility demands of SSGs (Davies et al., 2013). These SSGs involved elite AF players competing in a small-sided handballing game where the reduction in players resulted in small increases in total agility maneuvers (a maximum or near-maximum change of direction or deceleration to influence a contest). Although the only skill performed was handballing, an earlier soccer study using different constraints, found similar results (Duarte et al., 2009). Duarte et al. (2009) reported when there was a decrease in the number of soccer players from 4v4 to 2v2, with a pitch size of 20 m × 20 m, player intensity increased and more frequent tactical actions occurred. It was hypothesized this was due to more surface area being available per player. In contrast to these two studies, a rugby league study analyzing the effect of field size on the physiological and skill demands of players involved in SSGs, noted no significant skill involvement differences when using a 10 m × 40 m playing area versus a 40 m × 70 m playing area. Increases in distances covered at moderate, high and very high intensities, however, were noted in games played on the larger field size with senior elite players recording higher amounts than junior elite players (Gabbett et al., 2012a). When using SSGs as a way of assessing talent it is important the perimeter applied allows the skills in AF to occur naturally (i.e., kicking and handballing). It is therefore suggested the surface area per player should be representative of the surface area each player has during match-play.

The duration of each bout is an important consideration when discussing SSGs. Research has shown in a 3v3 soccer contest, bout duration did not have an effect on the number of technical actions performed per minute or proficiency (Fanchini et al., 2011). The authors did, however, note, as duration increased from 2 to 6 min there was a decrease in exercise intensity. Sampaio and Macas (2012) have suggested as players become more skilled they run less as their movement patterns are more intentional due to a greater tactical awareness of the game. When trying to emulate match-play intensity, 4-min bouts are suggested as the best choice. In an elite 4v4 soccer game, of 4 min in duration (with 3 min passive recovery), it was shown SSG intensity was comparable to generic aerobic interval training with the total distance covered per minute, total number of duels and lost ball possessions all being greater in the SSG than actual game play (Impellizzeri et al., 2006). Furthermore, manipulating the constraint of fewer ball touches (i.e., 1 or 2) increased the difficulty for players to perform technical actions making it more specific to match demands (Dellal et al., 2012).

Small-sided game play allows players more opportunities to gain possession of the ball to display their skill proficiency as well as more opportunities to apply game strategy and tactical maneuvers in an easily manipulated and convenient setting. This form of assessment replicates match-play conditions from an integrated physiological, tactical, and technical perspective. Considering player performance should be analyzed from within a simulated, competitive environment it appears SSG assessment is the best solution (other than actual match-play at Level-5) for assessing competition skill performance (Davids et al., 2013a). To effectively develop a small-sided game assessment, researchers could examine the notational analysis of match-play dynamics from the foundation stone. They could then apply these findings by modifying the SSG variables such as pitch area, the number of players participating, the rules by which the players are abiding by and the intensity at which the game is played. For example, a smaller number of players combined with a large pitch size will make players work at a higher exercise intensity (Hill-Haas et al., 2011).

Overall, Level 4 provides a more open playing environment along the continuum where the rules replicate match-play. The skill assessment will more closely resemble match-play assessment of their ability to not only obtain possession of the ball but deliver the ball under match like demands (i.e., pressure). This analysis could then be used in assessing player talent selection, player development tracking, the effectiveness of intervention programs and potentially how a player will perform during match-play. A limitation of Level-4 might be the reluctance of the coach to implement the game with contact (thereby reducing the representativeness of the assessment).

### Level-5: Match-Play

Match-play assessment resides at the highest point in the model, as this ultimately highlights a player’s ability to perform within the sport. When recruiters were interviewed as what they perceived as important for U13 soccer performance they identified the technical (i.e., first touch), tactical (i.e., decision making), and the psychological attributes (i.e., trainability) as being highly important, with other attributes such as physiological, anthropometrical, and sociological being less important suggesting recruiters apply a holistic multidisciplinary approach to talent selection (Larkin and O’Connor, 2017). This finding was supported in another soccer study interviewing eight Danish national team coaches (Christensen, 2009). Within this study it was highlighted how coaches regarded game intelligence (i.e., tactical awareness), peak competences (i.e., technical skill), willingness to learn, work ethic, and dedication as the most important qualities when selecting talented players. These studies highlight how match-play performance is a key component in the talent identification process in comparison to objective skill assessments.

It is common practice for AF coaches to select teams based off competition performance, however, the effectiveness of this method has had limited investigation in the literature. Black et al. (2016) identified how higher skilled players perform greater physical and technical performances following peak periods of match-play in comparison to lesser skilled players. When coaches subjectively rated player match performance, Johnston et al. (2012) found the higher skilled players had more kicks and disposals per minute, covered less distance and performed fewer high-speed efforts than lesser skilled players. This finding was supported in another AF study where skill performance, in comparison to physical activity, was found to be more important to a coaches’ perception of performance (Sullivan et al., 2014b). In contrast to these two studies, Johnston et al. (2016) investigated the relationship between movement demands, match events and match performance in AFL players. They noted how higher skilled players had higher match durations, covered greater total distance and spent more time running at high speeds per minute than lesser skilled players.

Combined, this research supports how match-play could be used as an integrated assessment. Unfortunately, there are limited opportunities for a player to be selected to play at the highest level and once selected there may be limited chances for the player to display their capabilities. For example, external variables such as weather, opposition tactics and flow of play may impact the amount of possessions a player has. Therefore, in an attempt to identify talent, an array of tests along the continuum may be required to more effectively assess specific components of performance. As such, it should be acknowledged a limitation of the current model is it does not consider other factors influencing talent detection and development (e.g., social, coaching, physical, physiological, and psychological) (Williams and Reilly, 2000; Pazo Haro et al., 2012). Therefore, the assessment of technical skill ability is just one piece of the talent identification conundrum.

## Conclusion

There are many factors to consider when implementing a technical skill assessment. The prediction of talent is more likely to be successful when tests are more representative of match-play and assessed within an integrated approach. The EPA model, which contains three stages – identification of key components, the assessment of these components and the acquisition of these components, has been combined with the RLD framework to review current Australian Football technical skill assessments and develop a structured framework for practitioners to consider when assessing, or developing, new assessments of technical game-based skills.

A 5-level performance assessment model has been proposed that explores the skill assessment continuum. As the tests apply the notational analysis and move from Level-1 (laboratory analysis) to Level-5 (match-play assessment) there is a step-wise progression in the performance demands and integration of the four components to more closely represent match-play conditions (representative design). For example, Level-1 can provide a detailed isolated analysis of the kicking action in a controlled and stable environment, the kicking action is pre-determined with no opposition pressure. Level-2 is also an isolated analysis, with no opposition pressure and delivering the ball to a pre-determined location. This level assesses the technical component of the kick in a field-based setting with the focus on skill proficiency. Level-3 is a more dynamic field-based assessment, involving the combination of several match-specific components at the one time (e.g., technical, physical, psychological). The constraints are more open (e.g., the ball needs to be passed to a moving teammate), however, there are no opposition present. Level-4 looks at integrating all of the components under similar performance demands in a field-based small-sided game. Normal game rules apply, and opponents are present, which allows for a greater assessment of match-play skill execution than technical competency. Match-play resides at Level-5 as this is the ultimate level of skill assessment, highlighting a player’s ability to perform in the sport. For example, a player’s ability to effectively dispose of the ball under scoreboard pressure or opposition tactics (e.g., playing area pressure). Assessments from these tests could be used in conjunction with each other to profile players, track player development and display player strengths whilst identifying specific areas of improvement along the AF talent pathway.

## Author Contributions

NB wrote the article. JB helped with the conceptual idea. KB and PL helped with the conceptual idea and the editing.

## Conflict of Interest Statement

The authors declare that the research was conducted in the absence of any commercial or financial relationships that could be construed as a potential conflict of interest.
